# Computing the Deflection of the Vertical for Improving Aerial Surveys: A Comparison between EGM2008 and ITALGEO05 Estimates

**DOI:** 10.3390/s16081168

**Published:** 2016-07-26

**Authors:** Riccardo Barzaghi, Daniela Carrion, Massimiliano Pepe, Giuseppina Prezioso

**Affiliations:** 1 Department of Civil and Environmental Engineering (DICA), Politecnico di Milano, Milan 20133, Italy; daniela.carrion@polimi.it; 2 Architecture, Built environment and Construction engineering (ABC), Politecnico di Milano, Mantova 46100, Italy; massimiliano.pepe@polimi.it; 3 Department of Science and Technology (DIST), University of Naples “Parthenope”, Naples 80143, Italy; pina.prezioso@uniparthenope.it

**Keywords:** deflection of the vertical, aerial surveys, EGM2008, Least Squares Collocation

## Abstract

Recent studies on the influence of the anomalous gravity field in GNSS/INS applications have shown that neglecting the impact of the deflection of vertical in aerial surveys induces horizontal and vertical errors in the measurement of an object that is part of the observed scene; these errors can vary from a few tens of centimetres to over one meter. The works reported in the literature refer to vertical deflection values based on global geopotential model estimates. In this paper we compared this approach with the one based on local gravity data and collocation methods. In particular, denoted by *ξ* and *η*, the two mutually-perpendicular components of the deflection of the vertical vector (in the north and east directions, respectively), their values were computed by collocation in the framework of the Remove-Compute-Restore technique, applied to the gravity database used for estimating the ITALGEO05 geoid. Following this approach, these values have been computed at different altitudes that are relevant in aerial surveys. The (*ξ*, *η*) values were then also estimated using the high degree EGM2008 global geopotential model and compared with those obtained in the previous computation. The analysis of the differences between the two estimates has shown that the (*ξ*, *η*) global geopotential model estimate can be reliably used in aerial navigation applications that require the use of sensors connected to a GNSS/INS system only above a given height (e.g., 3000 m in this paper) that must be defined by simulations.

## 1. Introduction

In recent years, the increasing use of sensors connected to a GNSS/INS system has led to deep studies on the impact of the Earth’s actual gravity field [1,2,3,4].

In particular, the effect of the deflection of the vertical [5] has been investigated. Such analyses have shown the importance of considering this signal both in terms of degradation of the accuracy [6] and in terms of improvement of the observations [7,8,9].

Additionally, the most important companies specialized in GNSS/INS positioning (e.g., Applanix or Novatel) suggested the necessity of using a proper (*ξ*, *η*) model in order to achieve high performance results.

In most of the works reported in literature, the deflection of the vertical components are computed at ground level, on the Digital Terrain Model (DTM). However, this approach is unsatisfactory when aerial applications are considered. As it is well known, the values of the deflection of the vertical vary with altitude [10], decreasing while moving away from the ground level.

Thus, in order to evaluate the impact of the deflection of the vertical in aerial surveys, it is important to define a rigorous approach to its calculation that takes into account its variation with altitude. Thus, proper estimation formulas must be considered that allow computing (*ξ*, *η*) in any given point in space, i.e.:

(1) $$\left\{ \begin{array}{l} \xi \;=\;f_{\xi }\left(\varphi ,\;\lambda ,\;h\right)\\ \eta \;=\;f_{\eta }\left(\varphi ,\;\lambda ,\;h\right) \end{array}\right.$$

In this paper, two different methods for estimating (*ξ*, *η*) are presented, i.e., collocation and spherical harmonic representation. The collocation approach based on gravity data will be revised and numerical results will be computed for some relevant cases.

These results will be then compared with the values obtained using the other proposed estimation method based on one of the most recent and detailed Global Geopotential Model (GGM), the EGM2008 [11].

## 2. Predicting the Deflection of the Vertical Using Gravity Data and the Collocation Method

**Figure 1:**
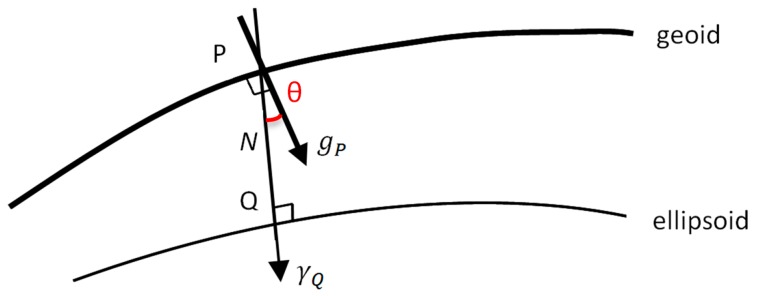
The deflection of the vertical at geoid level.

The deflection of the vertical is the difference in direction between the direction of Earth’s gravity vector and some reference direction, such as the direction perpendicular to a given reference ellipsoid or the direction of some reference gravity field (the normal gravity) [4]. Considering the normal to the ellipsoid as the reference direction, at the geoid surface we have the situation sketched in Figure 1, where the angle is exaggerated to explain more clearly the physical aspect of the problem.

**Figure 2:**
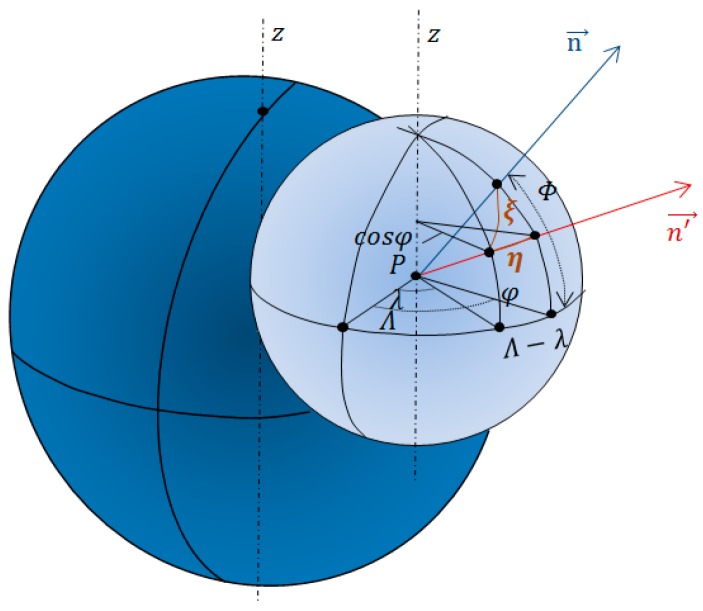
The deflection of the vertical components.

The deflection of the vertical vector can be decomposed into two mutually perpendicular components: the north-south or south (*ξ*), positive toward the north, and east-west or first vertical (*η*), positive in the east direction. These two components are represented in Figure 2 with respect to the unit sphere, centred at point *P*, having the *z* axis parallel to the Earth rotation axis, the equatorial circle parallel to the Earth’s equatorial plane and the reference meridian in a plane parallel to the Greenwich meridian. 

The two unit vectors $\vec{n}$ and ${\vec{n}}^{'}$ indicate, respectively, the vertical direction and the direction of the normal to the ellipsoid in a given point *P*.

They are functions of the astronomical (Φ, Λ) and geodetic (φ,λ), latitude and longitude of *P*, respectively:

(2) $$\{\begin{array}{c} \vec{n}=\left(\Phi ,\;\Lambda \right)\\ {\vec{n}}^{'}=\left(\varphi ,\lambda \right) \end{array}$$

In the so-called spherical approximation, the components *ξ* and *η* of the deflection of the vertical are given by [12]:

(3) $$\{\begin{array}{c} {\xi =\;-\frac{1}{r\cdot \gamma }\frac{\partial T}{\partial \varphi }|}_{P}\;\\ {\eta =\;-\frac{1}{r\cdot \gamma \cdot cos\varphi }\frac{\partial T}{\partial \lambda }|}_{P} \end{array}$$

where:

*γ*: normal gravity in *P*;
*ϕ*: latitude;
*λ*: longitude;
*r*: distance of *P* from the geocenter.

In Equation (4), *T* is the anomalous or disturbing potential defined as [10,13]:

(4) T(P)=W (P)−U (P)

As it is well known, *T* is a harmonic function in space outside the masses. The anomalous potential is related to the geoid undulation *N*, the distance between the geoid and the ellipsoid along the ellipsoid normal (Figure 1), by the Bruns’ formula:

(5) $$N\left(P\right)=\frac{T\;\left(P\right)}{\gamma }$$

Additionally, one can define the gravity anomaly as:

(6) Δg=g(P)−γ (Q)

i.e., the difference between the magnitude of the actual gravity in point *P* onto the geoid and the magnitude of the normal gravity in *Q* on the ellipsoid (Figure 1).

As for the components (*ξ*, *η)* of the deflection of the vertical, it can be proved that Δg is functionally related to the anomalous potential *T* by the following formula that holds in spherical approximation:

(7) $$\Delta g=\;-\frac{\partial T}{\partial r}-\frac{2}{r}T$$

These functions can be defined at ground level as well as in the space outside the Earth’s body. 

In order to evaluate the impact of the deflection of the vertical on GNSS/INS, applied, e.g., to photogrammetry, (*ξ*, *η)* are to be estimated at flight altitude using proper analytical methods. One possible methodology for doing so is to apply collocation to gravity data in the framework of the Remove-Compute-Restore (RCR) technique [14].

In the remove step, the long-wavelength component (provided by a Global Geopotential Model, GGM) and the short-wavelength component (considered in the Residual Terrain Correction effect, RTC) of gravity anomalies are removed from the original gravity data. Consequently, residual gravity anomalies are obtained as:

(8) $$\Delta g_{r}=\Delta g_{obs}-\Delta g_{GGM}-\Delta g_{RTC}$$

where:

Δg*_r_*: residual gravity anomalies;
Δg*_obs_*: observed gravity anomalies;
Δg*_GGM_*: contribution of a Global Geopotential Model;
Δg*_RTC_*: gravity anomalies correction due to terrain.

The residuals Δg*_r_* are then used as input values in the Least Squares Collocation (LSC) procedure to get the estimates of the residual deflection of the vertical components (*ξ_r_*, *η_r_*) [15]. The estimated values are given by:

(9) $$\left[\begin{array}{c} \xi_{r}\\ \eta_{r} \end{array}\right]=\left[\begin{array}{c} C_{\xi \Delta g}\\ C_{\eta \Delta g} \end{array}\right]{\left[C_{\Delta g\Delta g}+\sigma_{n}^{2}I\right]}^{-1}\left(\Delta g_{r}+n\right)$$

with:

$C_{\xi \Delta g}$ and $C_{\eta \Delta g}\;$: cross-covariances between (*ξ_r_*, *η_r_*) and Δg*_r_*;
$C_{\Delta g\Delta g}\;$: auto-covariance of Δg*_r_*;
*n*: noise in gravity;
$\sigma_{n}^{2}$: noise variance.

We can then obtain the total components of the vertical deflection in a given point in space by adding the (*ξ_GGM_*, *η_GGM_*) GGM signal and the (*ξ_RTC_*, *η_RTC_*) RTC effects (restore step). So, in the end, the final estimated values are expressed as:

(10) $$\{\begin{array}{c} \xi =\;\xi_{GGM}+\xi_{RTC}+\xi_{r}\\ \eta =\;\eta_{GGM}+\eta_{RTC}+\eta_{r} \end{array}$$

## 3. The Deflection of the Vertical by Spherical Harmonic Expansion: the EGM2008 GGM 

As stated above, one can compute the (*ξ_GGM_*, *η_GGM_*) components using a GGM. Many different models have been estimated and are currently used in geodetic applications. It is, thus, of interest to compare the GGM estimates with the corresponding values obtained by using the procedure detailed in the previous paragraph. Particularly, this will be done in the framework of the investigation proposed in this paper to test if the collocation based estimates can significantly improve the aerial survey with respect to the use of the GGM only.

To this aim, we considered one of the most accurate and high-frequency global geopotential models, i.e., EGM2008. This geopotential model is estimated as a combination of GRACE (Gravity Recovery and Climate Experiment) satellite data [16], a global gravity data grid and topographic data [17]. It is complete to spherical harmonic degree and order 2159 and contains additional coefficients to degree 2190 and order 2159.

**Figure 3:**
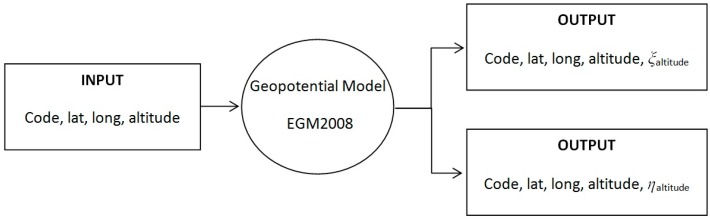
Flow chart of the algorithm for the point-wise estimation of *ξ* and *η*.

The EGM2008 has been validated, in the central Mediterranean [18], comparing gravity data and GPS/levelling data, while in Europe the model values were compared with those obtained by astrogeodetic measurements [19]. This comparison has provided residuals, in terms of the vertical deflection, of about 3 arc seconds (root mean square—RMS) that proves the high quality of this geopotential model (furthermore, in [20] studies on the accuracy and quality of the EGM2008 model in different parts of the world are described). At the NGA website the users can find the Fortran software (Figure 3) which allows computing the values of *ξ* and *η* in a given point in space, as well as the file (EGM2008_to2190_TideFree) associated with it, containing the spherical harmonics fcoefficients of the EGM2008 model. This file contains the fully normalized, unit-less, spherical harmonic coefficients $\left\{{\bar{C}}_{nm},\;{\bar{S}}_{nm}\right\}$ of the EGM2008 Earth’s gravitational potential and the associated (calibrated) standard deviations $\left\{\sigma_{{\bar{C}}_{nm}},\;\sigma_{{\bar{S}}_{nm}}\right\}$.

Thus, the model implying gravitational potential is described via the coefficients ${\bar{C}}_{nm}$, ${\bar{S}}_{nm}$, and can be derived as [21]:

(11) $$V\left(\varphi ,\lambda ,r\right)=\;\frac{GM}{r}\left[1+\overset{N_{max}}{\underset{n=2}{\sum }}{\left(\frac{a}{r}\right)}^{n}\overset{n}{\underset{m=0}{\sum }}\left({\bar{C}}_{nm}\cos m\lambda +{\bar{S}}_{nm}\text{sin }m\lambda \right){\bar{P}}_{nm}\left(\sin\varphi \right)\right]$$

where:

*ϕ*, *λ*, *r*: spherical geocentric coordinates;
*a*: semi-major axis of Earth;
*GM*: gravitational constant times mass of Earth;
*n, m*: degree and order of spherical harmonic;
${\bar{P}}_{nm}$: fully normalized Legendre functions;
${\bar{C}}_{nm}$, ${\bar{S}}_{nm}$: fully normalized coefficients.

In the EGM2008 model the scaling parameters {GM, a} have the following numerical values:

*a* = 6,378,136.3 m

*GM* = 3,986,004.415 × 10^8^

Thus, one can obtain, by spherical harmonic synthesis, the two vertical deflection components at any point on and outside the Earth as implied by this model. 

Using Equation (11) and the software mentioned above, we computed the model values to be used in the comparisons described in the following. 

## 4. Comparison between Vertical Deflection Models Derived by Collocation and EGM2008 in the Central Mediterranean Area

**Figure 4:**
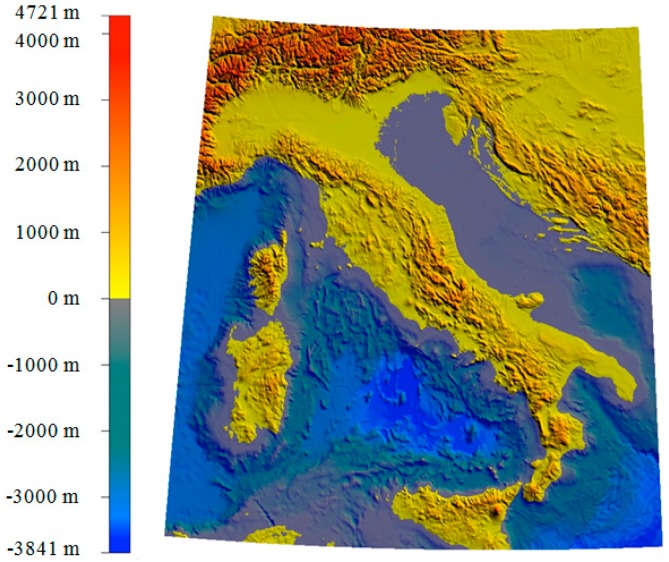
Topography (ETOPO1) of the study area.

The estimates, obtained by the RCR/LSC method and the EGM2008 model have been compared in the central Mediterranean area (37° N ≤ *ϕ* ≤ 47° N, 7° E ≤ *λ* ≤ 19° E) whose topography is shown in Figure 4 [22]. 

The gravity signal of this region has sharp variations due to the mountain ranges of the Alps and the Apennines, and to strong geophysical features (e.g., the Calabrian Arc). Thus, the comparison between the two estimates is particularly relevant due to the rough structure of the gravity field in the selected area. The deflection of the vertical components, computed by the RCR/LSC procedure, have been estimated starting from the Italian gravity database, the same used for estimating the ITALGEO05 geoid [23]. This gravity database consists of about 440,000 gravity values that are quite homogeneously distributed in the estimation area. 

In order to account for the low-frequency features of the gravity field, the EIGEN-6C3 model [24] to degree and order 1000 was considered. The residual terrain effect has been computed using the 3″ SRTM DTM [25] with respect to a mean elevation surface obtained by filtering the detailed DTM with a 10′ moving average window which has been optimized with respect to the GGM used in the remove step.

The GGM and terrain effects have been, subsequently, subtracted from the gravity data and the reduced gravity values have been then gridded on a 2*′* × 2*′* resolution grid by LSC [23]. 

Based on this gravity grid, the vertical deflection residuals were computed, by RCR/LSC, using the GRAVSOFT package (DTU, Copenhagen, Denmark) [26], on the same 2*′* × 2*′* grid, at different altitudes, namely 1000 m, 2000 m, 3000 m, 4000 m, 5000 m, and 7000 m.

**Figure 5:**
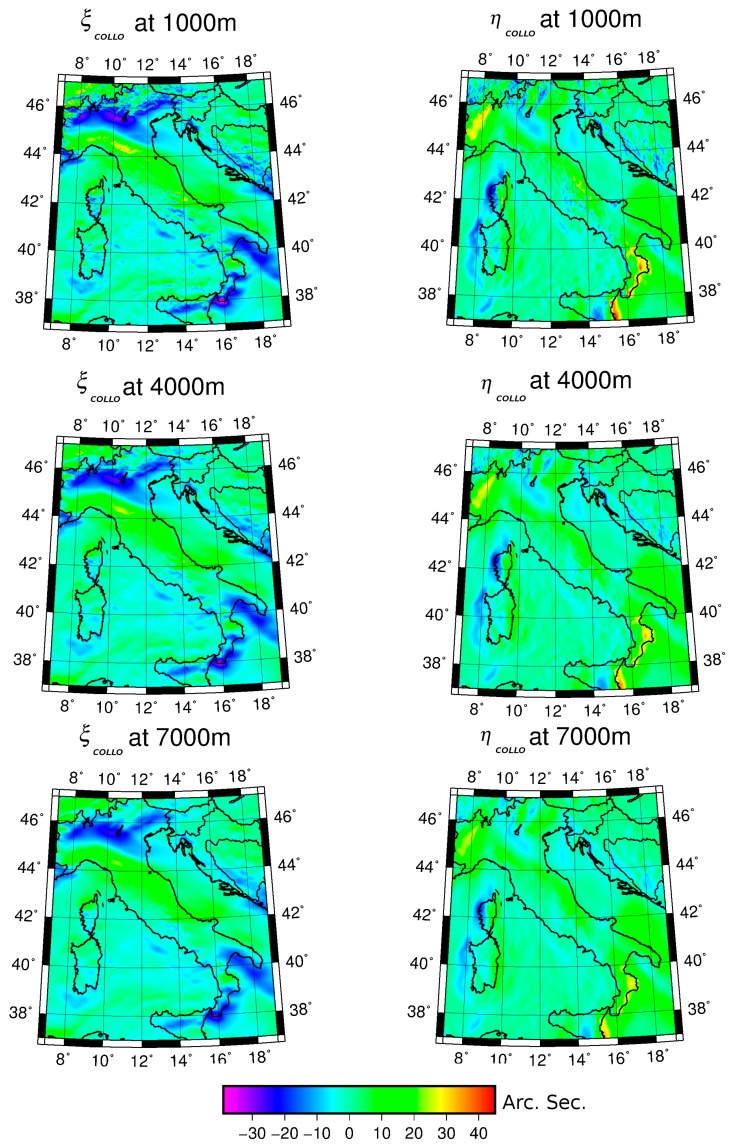
Vertical deflection values, in arc-seconds, at 1000, 4000, and 7000 m altitudes.

The model and the RTC vertical deflection components were then added, in the same points, for each selected scenario. The final estimates (*ξ_COLLO_*, *η_COLLO_*), at different altitudes, are shown in Figure 5. 

As expected, the estimated values are smoother while increasing the grid height. Table 1 contains the statistics of the model, RTC, and residual components at the three different altitudes.

The analysis of these statistics highlights that the component related to terrain decreases more rapidly than the other components.

At 1000 m, the contribution of the topography is relevant, having values comparable to those of the geopotential model.

At an altitude of 4000 m a significant reduction of the terrain component occurs since the related values are about half than those obtained at 1000 m altitude.

At 7000 m, the terrain component is about one fourth of the 1000 m effect, both in minimum and maximum values. In particular, the vertical deflection components, relative to the contribution of the terrain (short wavelength) assume values close to zero, and minimum and maximum values of a few seconds.

On the contrary, the model contribution is smoothly decreasing with altitude and the same holds, even though to a less extent, for the residual component.

Thus, from this analysis it can be concluded that the model component gives the main contribution at high altitude while the RTC is relevant when approaching the ground level. So, it can be argued that the impact of the high-frequency terrain effect on navigation sensors could be significant mostly at low flight altitude. 

Subsequently, the vertical deflection values (*ξ_GGM_,* *η_GGM_*), obtained by the EGM2008 geopotential model, were estimated on the same grids and compared with those of the ITALGEO05 model (*ξ_COLLO_, η_COLLO_*), thus giving the discrepancies:

(12) $$\{\begin{array}{c} \Delta \xi =\;\xi_{COLLO}-\xi_{EGM2008}\\ \Delta \eta =\;\eta_{COLLO}-\eta_{EGM2008} \end{array}$$

Statistics of these discrepancies are shown in Table 2.

The discrepancies are smaller and smaller while increasing the grid height since the two estimates of the deflection of the vertical become smoother. This behaviour is coherent with the results of Table 1 and is physically consistent being that the gravity field is more regular at larger distances by the causative masses. 

To understand the impact of the two different estimates of the deflection of the vertical on GNSS/INS instruments in aerophotogrammetric applications, we computed the horizontal and vertical position errors induced by neglecting these discrepancies. As proved in [9], the *δh* error in the horizontal position and the *δv* error in the vertical position, introduced neglecting the deflection of the vertical (*DOV*), are given by:

(13) δh=H sin(DOV)

(14) $$\delta v=H\;tg\left(\frac{FOV}{2}\right)sin\left(DOV\right)$$

where *H* is the flight altitude, *FOV* is the camera field of view and *DOV* is the component of deflection of the vertical in the vertical plane orthogonal to the flight direction. The *DOV*, in a given direction having azimuth *α*, is related to the (*ξ,* *η*) component by the formula [10]:

(15) DOV=ξ cosα+η sinα

Thus, in order to evaluate the maximum difference in *(δh, δv)* induced by the difference between the *DOVs* computed using the collocation procedure and the EGM2008 GGM, one can compute:

(16) ΔDOV=Δξcosα+Δη sinα

and, for each grid knot, find the maximum of:

(17) Δ(δh)≅H⋅ΔDOV

(18) $$\Delta \left(\delta v\right)≅Htg\left(\frac{FOV}{2}\right)\cdot \left(\Delta DOV\right)$$

with respect to *α* (in the reasonable hypothesis that sin(ΔDOV)≅ΔDOV).

This computation has been carried out at the three different flight altitudes used in computing the (*ξ, η*) values and for two different *FOV* angles, 45° and 70°. 

The results are summarized in Table 3 where the maxima of the absolute values of *Δδh* and *Δδv* are listed for the considered cases.

**Figure 6:**
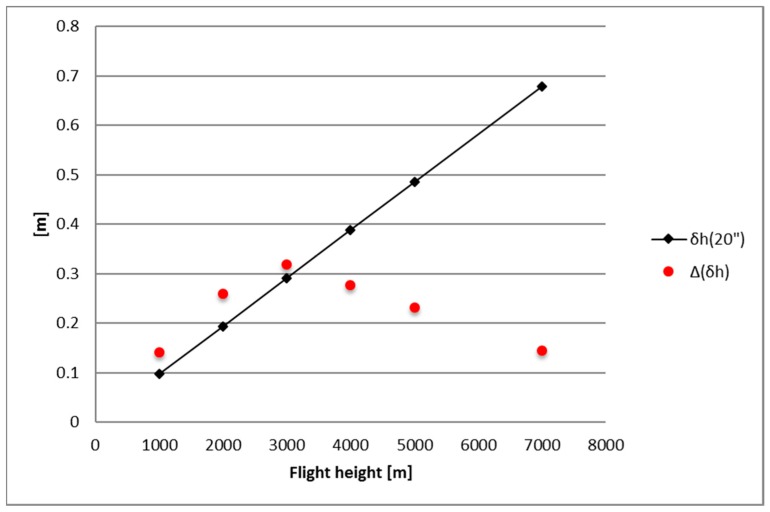
Values of *δh* based on *DOV* = 20”compared to Δ*δh* at different flight altitudes.

**Figure 7:**
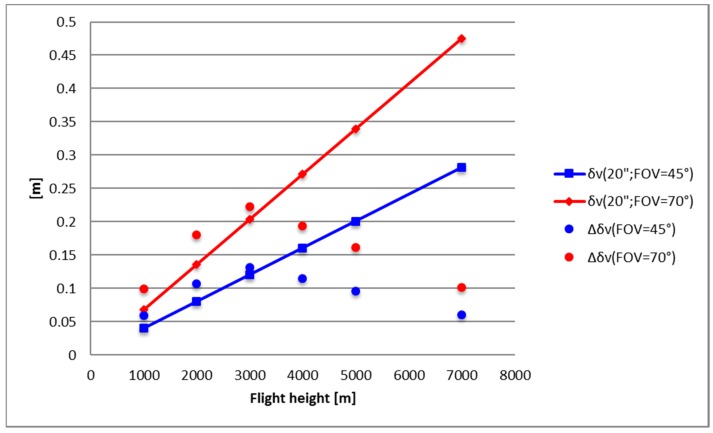
Values of *δv* based on *DOV* = 20” compared to Δ*δv* at different flight altitudes and different *FOV* angles (45°–70°).

At flight altitude higher than 3000 m these values are quite small if compared with the full *δh* and *δv* effects (Formulas (13) and (14)) obtained using *DOV* = 20” (see Figure 6 and Figure 7 and Table 4). According to [9], this can be considered the value above which the position errors *δh* and *δv* become critical and corrections for the DOV should be taken into account.

On the contrary, up to 3000 m in flight altitude, the values in Table 3 are higher than those in Table 4 and, thus, the discrepancies (*Δδh*, *Δδv)* are of the same order of magnitude of the vertical and horizontal errors. As already pointed out, this is a direct consequence of the higher discrepancies between the collocation and the EGM2008 estimates of (*ξ*, *η*). 

Thus, based on our computations, (*ξ_COLLO_, η_COLLO_*) should be used up to 3000 m in flight altitude, given that the RCR/LSC procedure is a more refined method able to better reproduce high-frequency details.

## 5. Conclusions

Two different estimates of the deflection of the vertical have been compared to assess their impact on aerophotogrammetry. This has been done in the central Mediterranean area where (*ξ,* *η*) have been computed using EGM2008 GGM and RCR/LSC based on local gravity data. This test is quite relevant since, in the selected area, the gravity field is rough due to the strong gravity signatures coming from the rugged topography of the Alps and the Apennines and the geodynamic signals present in the region (e.g., the Ivrea Body). In terms of the impact on navigation systems, the discrepancies between the two estimates proved to be relevant up to 3000 m in flight altitude. Below this altitude, the differences in the two estimates lead to horizontal and vertical errors that are larger than critical values defined in [9]. This is mostly related to the topographic signal that is not properly modelled by the EGM3008 model. Thus, in this case, in order to effectively correct for these distortion effects, it should be required to estimate (*ξ,* *η*) through the more complex RCR/LSC procedure based on local gravity data. As the flight altitude increases, the discrepancies between the EGM2008 model estimate and the one based on RCR/LSC become smaller and smaller. As a consequence, the errors in vertical and/or horizontal positions implied by the differences between the two estimates can be neglected since they are smaller than the critical values given in the literature. Hence, based on the results in this paper, above 3000 m in flight altitude, in aerophotogrammetry and remote sensing applications, the deflection of the vertical values estimated based on EGM2008 can be safely used for improving the position and the attitude of the sensors.

## Figures and Tables

**Table 1:** RCR/LSC procedure: statistics of vertical deflection values at 1000, 4000, and 7000 m altitudes.

**1000 m**						
	**Geopotential Model Component (EIGEN-6C3)**		**Residual Component**		**Terrain Component**	
	***ξ* (*"*)**	***η* (*"*)**	***ξ* (*"*)**	***η* (*"*)**	***ξ* (*"*)**	***η* (*"*)**
**Mean**	−1.920	2.004	0.204	0.042	−0.007	0.007
**Standard Deviation**	7.588	6.998	1.286	1.014	2.931	2.654
**Min**	−39.123	−29.447	−9.4459	−6.5066	−28.2366	−24.4967
**Max**	28.991	39.688	8.6725	5.8911	32.2476	21.8262
**4000 m**						
	**Geopotential Model Component (EIGEN-6C3)**		**Residual Component**		**Terrain Component**	
	***ξ* (*"*)**	***η* (*"*)**	***ξ* (*"*)**	***η* (*"*)**	***ξ* (*"*)**	***η* (*"*)**
**Mean**	−1.905	−1.983	0.200	0.041	−0.006	0.004
**Standard Deviation**	6.192	6.370	0.965	0.764	1.517	1.250
**Min**	−33.768	−24.727	−6.723	−4.724	−15.062	−14.141
**Max**	25.411	35.244	5.939	4.261	17.658	15.280
**7000 m**						
	**Geopotential Model Component (EIGEN-6C3)**		**Residual Component**		**Terrain Component**	
	***ξ* (*"*)**	***η* (*"*)**	***ξ* (*"*)**	***η* (*"*)**	***ξ* (*"*)**	***η* (*"*)**
**Mean**	−1.891	1.963	0.197	0.042	−0.005	0.004
**Standard Deviation**	6.368	5.866	0.760	0.611	0.907	0.697
**Min**	−29.512	−20.946	−4.8791	−3.5069	−7.9262	−5.6898
**Max**	22.443	31.604	4.1568	3.3674	7.8965	6.1959

**Table 2:** Statistic of (*Δξ*, *Δη*) values at different altitudes.

	**1000 m**		**2000 m**		**3000 m**	
	***Δξ* (*"*)**	***Δη* (*"*)**	***Δξ* (*"*)**	***Δη* (*"*)**	***Δξ* (*"*)**	***Δη* (*"*)**
**Mean**	0.199	0.065	0.198	0.063	0.195	0.063
**Standard Deviation**	2.088	2.055	1.622	1.607	1.109	1.099
**Min**	−22.946	−20.088	−20.390	−19.005	−15.667	−16.617
**Max**	21.858	28.769	19.148	26.360	13.539	21.816
	**4000 m**		**5000 m**		**7000 m**	
	***Δξ* (*"*)**	***Δη* (*"*)**	***Δξ* (*"*)**	***Δη* (*"*)**	***Δξ* (*"*)**	***Δη* (*"*)**
**Mean**	0.195	0.062	0.194	0.062	0.192	0.061
**Standard Deviation**	0.789	0.764	0.626	0.597	0.483	0.454
**Min**	−12.166	−10.902	−7.899	−6.000	−3.526	−2.411
**Max**	8.600	14.306	5.134	9.566	3.251	4.269

**Table 3:** Maximum absolute values of *Δδh* and *Δδv* at different altitudes.

	**1000 m**		**2000 m**		**3000 m**	
**Max |*Δδh*| (m)**	0.142		0.259		0.318	
**Max |*Δδv*| (m)**	***FOV* = 45°**	***FOV* = 70°**	***FOV* = 45°**	***FOV* = 70°**	***FOV* = 45°**	***FOV* = 70°**
	0.059	0.099	0.107	0.181	0.132	0.223
	**4000 m**		**5000 m**		**7000 m**	
**Max |*Δδh*| (m)**	0.277		0.232		0.145	
**Max |*Δδv*| (m)**	***FOV* = 45°**	***FOV* = 70°**	***FOV* = 45°**	***FOV* = 45°**	***FOV* = 45°**	***FOV* = 45°**
	0.115	0.194	0.096	0.162	0.060	0.102

**Table 4:** Absolute values of *δh* and *δv* at different altitudes based on *DOV* = 20”.

	**1000 m**		**2000 m**		**3000 m**	
**|*δh*| (m)**	0.097		0.194		0.291	
**|*δv*| (m)**	***FOV* = 45°**	***FOV* = 70°**	***FOV* = 45°**	***FOV* = 70°**	***FOV* = 45°**	***FOV* = 70°**
	0.040	0.068	0.080	0.136	0.120	0.204
	**4000 m**		**5000 m**		**7000 m**	
**|*δh*| (m)**	0.388		0.485		0.679	
**|*δv*| (m)**	***FOV* = 45°**	***FOV* = 70°**	***FOV* = 45°**	***FOV* = 70°**	***FOV* = 45°**	***FOV* = 70°**
	0.161	0.272	0.201	0.339	0.281	0.475

## Abbreviations

The following abbreviations are used in this manuscript:

ITALGEO05	Italian Geoid
EGM2008	Earth Gravitational Model 2008
GNSS/INS	Global Navigation Satellite System/Inertial Navigation System
DTM	Digital Terrain Model
GGM	Global Geopotential Model
RCR	Remove-Compute-Restore
RTC	Residual Terrain Correction
GRACE	Gravity Recovery and Climate Experiment
GPS	Global Positioning System
RMS	Root Mean Square
NGA	National Geospatial-Intelligence Agency
EIGEN-6C3	European Improved Gravity model of the Earth by New techniques
SRTM	Shuttle Radar Topography Mission
LSC	Least Squares Collocation
DOV	Deflection Of the Vertical
FOV	Field Of View
